# Read across for the derivation of Indoor Air Guidance Values supported by PBTK modelling

**DOI:** 10.17179/excli2018-1734

**Published:** 2018-11-05

**Authors:** Thomas Schupp

**Affiliations:** 1 Muenster University of Applied Science, Chemical Engineering, Stegerwaldstrasse 39,D-48565 Steinfurt, GERMANY

**Keywords:** indoor air, polyurethane, read across, PBTK modelling, VOC, metabolism

## Abstract

Polyurethane Flexible Foams (PUF) are versatile materials used in upholstered furniture and bed mattresses. Due to the production procedure, fresh foams emit volatile organic compounds (VOC) which may contribute to indoor air exposure. To evaluate the risk for consumers, the VOC concentration measured in chamber tests can be matched against existing benchmarks for indoor air like “Richtwerte” (RW) of the German UBA (Umweltbundesamt), “Lowest Concentration of Interest” (LCI) for construction products or derived no effect levels (DNEL) for consumer inhalation exposure. In a previous paper a method for the derivation of Indoor Air Guidance Values (IAGV) for VOC without RW, LCI or DNEL was developed. The method described made use of a sufficient toxicological database. For substances with an insufficient database, read across to structural analogues is a way forward to estimate Indoor Air Guidance Values (IAGV). In this work a read across exercise, supported by an open source physiology based toxicokinetic (PBTK) modelling program is demonstrated. The use of enzyme kinetic data for phase I and phase II metabolism is discussed and areas for further work were identified. For two substances with very limited toxicological data, allyloxypropanol (isomer mixture of 1-allyloxy-2-propanol and 2-allyloxy-1-propanol) and 2,3-di-ethyl-2,3-dimethylsuccinodintrile, Tentative Indoor Air Guidance Values of 750 µg/m³ and 65 µg/m³ were derived.

## Introduction

In the late 1990's the European Association of Flexible Polyurethane Foam Blocks Manufacturers (EUROPUR) startet to perform VOC emission tests for flexible polyurethane foams. This activity was introduced as part of a responsible care program to identify potentials for product improvement, and to address any health concerns consumers may have with regards to volatile organic compounds (VOC). Selected examples of PUF types were submitted to emission testing according to ISO 16000. Measurement results and a toxicological evaluation were published by Hillier et al. (2003[16]). Based on these experiences, VOC emission testing in chamber tests became part of EUROPURs product label CERTIPUR^®^ (EUROPUR, 2018[14]). Since 2003, many data concerning foam emissions were generated. These VOCs were matched against RW-values of the Advisory Group for Indoor Air Guidance Values of the German UBA (Ausschuss für Innenraumrichtwerte des Umweltbundesamtes) (UBA, 2012[24]), “Lowest Concentration of Interest” (LCI) (Joint Research Center, 2013[18]) or “Derived No Effect Level” (DNEL) for consumers under the REACh regulation (ECHA, 2012[12]). For some VOC, having repeated dose toxicity data but no RW-, LCI-values or DNELs, Indoor Air Guidance Values (IAGV) or Tentative Indoor Air Guidance Values (TIAGV) were derived (Schupp, 2018[21]). 

**Figure 1:**
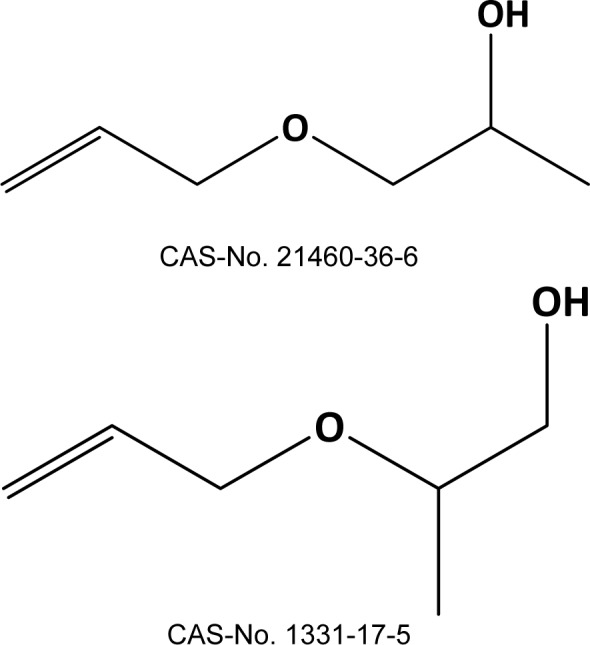
allyloxypropanol, both isomers

**Figure 2:**
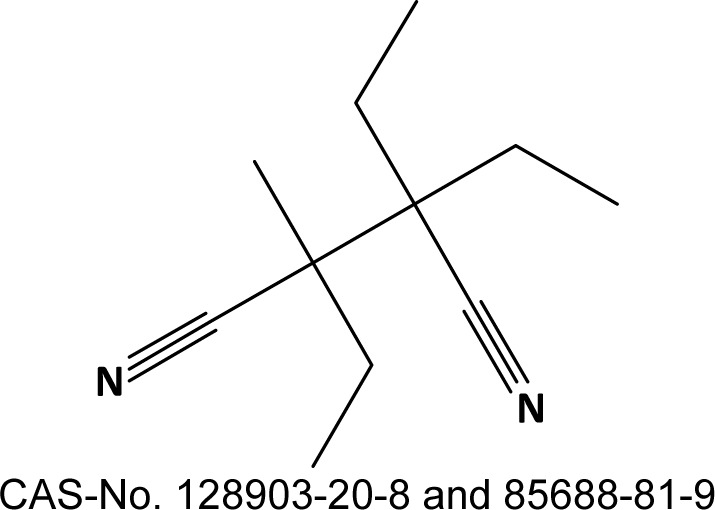
2,3-diethyl-2,3-dimethyl-succinodinitrile

For two VOCs that may be emitted from flexible polyurethane foam - allyloxypropanol (AOP, Figure 1(Fig. 1)) and 2,3-diethyl-2,3-dimethyl-succinodinitrile (DEDMSD, Figure 2(Fig. 2)) - repeated dose toxicity data are not available. This document focusses on the derivation of IAGV based on read across. 

## Methods

The procedure to derive Indoor Air Guidance Values (IAGV) is described in Schupp (2018[21]). Briefly, based on a repeated dose NOAEL/NOAEC or BMDL_5_, time scaling is applied for linear extrapolation of exposure time per day and per week. Subacute and subchronic studies are extrapolated to chronic exposure by default factors of 6 or 2, respectively. Interspecies extrapolation was split in a toxicokinetic factor (scaling for mg/kg body weight data) and a toxicodynamic factor (2.5 for remaining inter-species differences, or 1 of the most sensitive study out of otherwise equivalent datasets was chosen). Intra-species extrapolation for the general population was addressed by a factor of 10, and a further factor of 2 may be applied to cover higher breathing rates of children, if deemed appropriate.

For allyloxypropanol (AOP) and 2,3-diethyl-2,3-dimethyl-succinodinitrile (DEDMSD), structural analogues were searched by using the OECD QSAR toolbox v4.2 (OECD, 2018[20]). For read across purposes it is generally accepted that the toxic action of a compound is driven by its concentration over time in the target tissue - the toxicokinetic factor - and its specific interaction with the target tissue - the toxicodynamik factor. The toxicokinetic factor can be addressed by physiology based toxicokinetic modelling (PBTK). The open source software IndusChemFate v2.0 was used (Jongeneelen and ten Berge, 2011[19]). To run this program, substance data like molecular weight, density, vapour pressure, water solubility and octanol-water partition coefficient (Log Kow) are required. Missing data were calculated with the EPISUITE software v4.1 (EPA, 2017[13]). Metabolites were estimated with the OECD QSAR Toolbox software v4.2 (OECD, 2018[20]). Enterohepatic circulation was set to zero, renal re-absorption was set to “unknown”, and man at light work was modelled. For modelling metabolism, K_m_ and V_max_ values can be introduced, if available. In this work, lung (port of entry) and liver are assumed to be the only organs with relevant metabolism. For small VOCs, the CYP 450 2E1 is expected to be of most importance (Bolt et al., 2003[4]). For phase I metabolism, the potential range on enzyme activity shall be estimated. For DEDMSD, only phase I metabolism was considered. For AP, phase I metabolism (CYP) may create an oxirane structure (epoxide) which is expected to add to biological macromolecules. Therefore, phase II metabolism by epoxyhydrolases (EH) and glutathione-S-transferase (GST) as mitigation pathways for epoxide was calculated as well.

## Results

### Parameters for PBTK modelling

For the use of the physiological based toxicokinetic model the selection of V_max_ and K_m_ values for hepatic metabolism is crucial. Substance specific data for allyloxypropanol and 2,3-diethyl-2,3-dimethyl-succinodinitrile were not available. A literature search in TOXLINE for CYP 2E1 activity in human liver delivered data for 15 different organic compounds (supplementary material). Of these 15 componds, the first, second (median) and third quartile of enzyme activities are represented in Table 1(Tab. 1).

For human epoxide hydrolase (EH) and glutathione-S-transferase (GST) activity in liver, and CYP, EH and GST activity in lung, only data from Csanady et al. (1994[7], 2003[6]) were used (Table 2(Tab. 2); References in Table 2: Csanady et al., 1994[7], 2003[6]). The initial concentrations of GSH in liver and lung are 5.9 and 1.95 mM, respectively, and the zero order production rate of GSH in both liver and lung is 0.9 and 0.3 mM/h/kg, respectively. Results of the PBTK modelling are reported in the following chapters in combination with the substances under investigation.

### Allyloxypropanol (AOP)

Toxicological data for allyloxypropanol (AOP) are very limited: the acute oral LD_50_ in rat is 511 mg/kg; AOP is said to be irritating to the eye, but not the skin of rabbits (Propylene glycol allyl ether, 2018[5]). 

**Figure 3:**
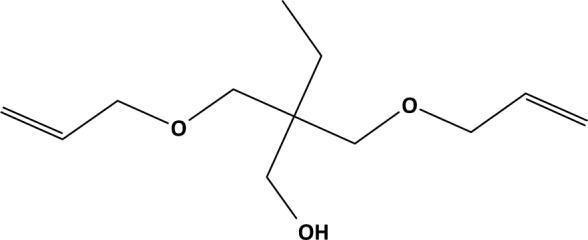
2,2-Bis(Allyloxymethyl)btane-1-ol (BAB, 2018; CAS-No. 682-09-7)

**Figure 4:**
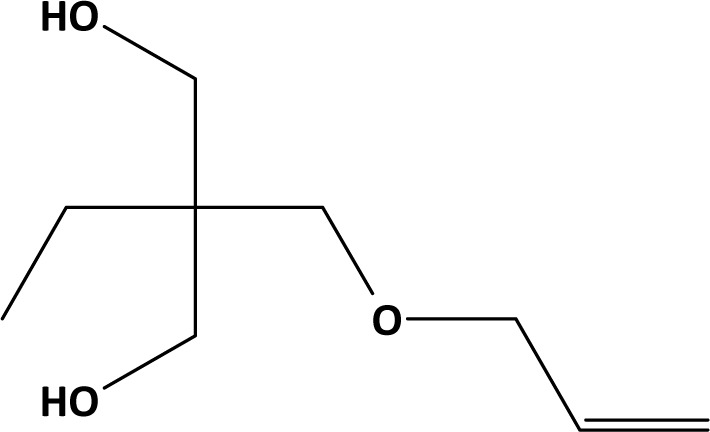
2-(Allyloxymethyl)-2-ethyl-propane-1,3-diol (AEPD, 2018; CAS-No. 682-11-1).

The QSAR Toolbox was used to search for structural analogues, representing hydroxylated aliphatic allyl ethers without other functional groups (QSAR Toolbox, 2018[20]). 2,2-bis(allyloxymethyl)butane-1-ol (BAB, CAS-No. 682-09-7, Figure 3(Fig. 3)) (BAB, 2018[3]), 3-Allyloxypropane-1,2-diol (APD, CAS-No. 123-34-2) (APD, 2018[2]) and 2-allyloxymethyl-2-ethyl-propanediol (AEPD, CAS-No. 682-11-1, Figure 4(Fig. 4)) (AEPD, 2018[1]) were identified as belonging to this class of compounds, having some toxicity data available. Oxidation by CYP 450 mono-oxygenases may transform these compounds as well as AOP to ketones, aldehydes and epoxides, as predicted by the OECD QSAR Toolbox v4.2 (supplementary material). Therefore, these substances were regarded as best candidates for the read across exercise to AOP.

In a subacute gavage study five rats per gender and dose received a daily dose of 0, 50, 200 or 800 mg/kg body weight BAB. Histopathology was confined to heart, spleen, liver, kidneys and adrenals. At the top dose, males had increased liver weights, biliary hyperplasia and cholangitis. The NOAEL was 200 mg/kg (BAB, 2018[3]).

Ten male and female rats received daily doses of 0, 50, 200 or 800 mg/kg BAB in corn oil for 90 days. At 200 and 800 mg/kg body weight, males showed dose-related significantly reduced body weight. In the high dose group haemoglobin concentration was reduced; urinary volume was increased and the urine pH decreased. Concerning hepatotoxicity, centrilobular hypertrophy was observed in males at 200 mg/kg; inflammatory cell infiltration and centrilobular inflammation were observed in the high dose groups. The NOAEL was 200 mg/kg/d (BAB, 2018[3]).

In a subacute gavage study, five male and female rats per dose received a daily dose of 0, 8, 40 or 200 mg/kg BW of AEPD. At the high dose, animals showed transient hypoactivity and salivation. At the top dose, haematology parameters were slightly elevated, as well as calcium and glucose in the blood, which was attributed to slight dehydration. Urinary status, body weight, organ weights and histopathology did not reveal adverse effects. Histopathology was not performed. Due to hypoactivity in the top dose group, the authors concluded a NOEL of 40 mg/kg/d (AEPD, 2018[1]). 

AEPD and BAB were negative in the bacterial reverse mutation assay, and BAB did not induce point mutations in the mouse lymphoma assay, but showed clastogenic activity. BAB was negative in the in vivo micronucleus assay in mice after i. p. administration; however, from the data accessible it is not quite clear whether the bone marrow was reached by the test substance. With the exemption of strain TA 1535 without metabolic activation, APD was negative in the bacterial reverse mutation assay (APD, 2018[2]; BAB, 2018[3]). 

BAB is not a teratogen, and histopathology in the sub-chronic study did not reveal adverse effects in reproductive organs (BAB, 2018[3]). 

As BAB has data from a sub-acute as well as from a sub-chronic study, and because this substance is a carrier of two allyloxy-equivalents per molecule, and because it has a nearly equivalent allyloxy group per weight ratio as AOP, it was selected for read-across. PBTK modelling was run for AOP and BAB, using the median for CYP activity. Physical chemical data used for PBTK modelling are summarized in Table 3(Tab. 3) (Reference in Table 3: BAB, 2018[3]).

**Figure 5:**
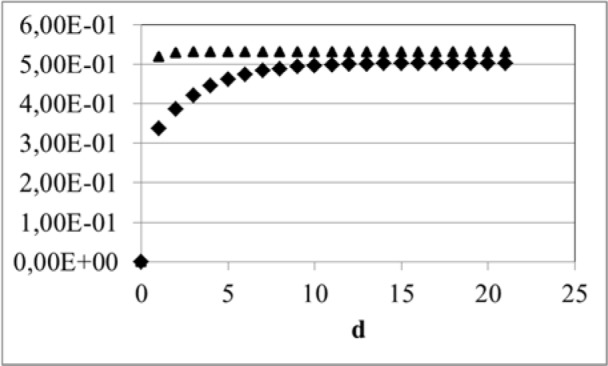
Liver concentration [µmol/L] of BAB (rhombus) and AOP (triangle) following continuous air exposure against 1 mg/m³

As is shown in Table 4(Tab. 4) AOP and BAB achieve almost equivalent concentrations in the target organ liver. This conclusion is valid for equal CYP activities for both substrates. Within about 3 days, AOP achieves a steady state copncentration whereas for BAB this is the case after about 11 days of continuous exposure (Figure 5(Fig. 5)).

If CYP 450 is more active against BAB than AOP, the picture changes. For example, assume that CYP 450 shows an activity against BAB at the 3^rd^ quartile - V_max_/K_m_ = 40.26 h^-1^ - , but against AOP at the 1^st^ quartile, only (V_max_/K_m_ = 8.73 h^-1^, Table 1(Tab. 1)). Against an exposure of 1 mg/m³ BAB, the steady state concentration in the liver then is 0.121 µmol/L; AOP results in the same steady state concentration if its air concentration is about 0.2 mg/m³, 5 times lower than that of BAB. The outcome is shown in Table 5(Tab. 5).

When not the parent compound but the first metabolite is critical for organ toxicity, physico-chemical data and metabolism data have to be introduced for the metabolites. For AOP, the epoxydation product is hydroxypropyl-glycidylether (HPGE), and epoxidation of BAB generates 2-{[(oxiran-2-yl)methoxy]-methyl}-2-{[(prop-2-en-1-yl)oxy]methyl}-butan-1-ol (BAB epoxide). Physical chemical data and metabolism data used for the PBTK modelling are summarized in Table 3(Tab. 3). Now the worst case assumption is rapid phase I metabolism for AOP and a slow one for BAB (3^rd^ quartile against 1^st^ quartile CYP 450 activity, Table 1(Tab. 1)). For the AOP epoxide and BAB epoxide, approximated V_max_^#^ and K_m_^#^ for liver and lung are introduced in the model, assuming that the concentration of GSH in lung and liver is not reduced by the exposure (for details see supplementary material). As in reality the level of GSH may become exhausted - which would finally end up in some cell damage and would not be in concordance with a NOAEL - the modelling was also run under assumption that only epoxide hydrolase serves for the decay of epoxides. Results are shown in Table 6(Tab. 6). 

The slower phase I metabolism of BAB compared to AOP results in its about five fold higher levels in the liver; decay of the formed epoxides by EH alone, or by EH and GST in combination leads to nearly equivalent levels of AOP and BAB epoxides in the liver. 

Results of the PBTK modelling will now be used for the read across from BAB to AOL. The oral NOAEL of BAB from the sub-chronic rat study was taken forward for the derivation of a Tentative Indoor Air Guidance Value (TIAGV) for AOP. If the kinetic parameters for phase I metabolism in the liver for both, AOP and BAB, are at the median (Table 1(Tab. 1)), both compounds result in similar concentrations in the target organ liver. The epoxides of AOP and BAB would not result in remarkably different levels in the liver even under a worse case assumption of rapid epoxidation of AOP and slow epoxidation of BAB. Therefore, the toxicokinetic read-across factor is 1. Other extrapolation factors are selected following the IAGV scheme published previously (Schupp, 2018[21]). The study duration factor is 1 as sub-acute and sub-chronic studies delivered the same NOAELs and LOAELs for BAB; therefore, it is not expected that a further extension of exposure would generate lower NOAELs and LOAELs. As fertility is covered by histopathology of reproductive organs in the sub-chronic study only, a factor of 2 is applied for reduced sensitivity when compared to a 1-generation study. All these data points now allow to derive an indoor air guidance value:

NOAEL_sub-chronic_ 200 mg/kg/d

sub-chronic to chronic :1

PBTK scaling :1 

oral to inhalation :2

fertility :2

inter-species :10

intra-species :10

children: :2

NOAEL_ consumer_ 0.25 mg/kg/d.

With 60 kg body weight (x 60 kg) and 20 m³ breathing volume per day (:20 m³/d), the tentative indoor air guidance value is

TIAGV 750 µg/m³.

### 2,3-Diethyl-2,3-dimethyl-succinodinitrile (DEDMSD)

Substance specific data for DEDMSD were not identified. For read across, data for aliphatic tertiary nitriles were searched with the OECD QSAR Toolbox v4.2 (OECD, 2018[20]). 2,2,3,3,-tetramethyl-succinodinitrile (TMSD, CAS-No. 3333-52-6) and 2,2,4-trimethyl-4-phenyl-butane nitrile (TMPBN, CAS-No. 75490-39-0) (TMPBN, 2018[23]) could be identified as structural analogues with repeated dose toxicity data. For TMSD a TIAGV was derived previously (Schupp, 2018[21]). To focus the search on tertiary nitriles is justified as primary and secondary nitriles may undergo alpha-hydroxylation; the resulting cyano-hydrins can easily release cyanide ions, which is not the case for the tertiary nitriles. For example, sodium thiosulfate serves as a sulfur-depot for detoxification of cyanide by supporting its transformation to thiocyanate; sodium thiosulfate was protective for intoxication with adiponitrile, but not for TMSD (Doherty et al., 1982[11]). 

In a 28 d gavage study, rats received daily doses of 0, 15, 150 or 500 mg TMPBN per kg body weight per day; the mid and high dose caused significant increased liver weights in males and females accompanied with centrilobular liver enlargement which was not reversible in the 14 d post exposure observation high dose group. High dose group males showed eosinophilic infiltrations in kidney tubular cells. The authors concluded on a NOAEL of 150 mg/kg/d (TMPBN, 2018[23]).

In subchronic oral studies vof TMSD with rats and dogs, the NOAEL was 1 mg/kg b.w. and 3 mg/kg was the LOAEL in both species. Kidneys, but also livers were target organs (DFG Tetramethylsuccinnitril, 2001[9]). 

TMPBN was negative in the bacterial reverse mutation assay and in the cytogenicity assay with human lymphocytes with and without metabolic activation (TMPBN, 2018[23]). Tetramethyl-succinodinitrile (TMSD) was negative in the bacterial reverse mutation assay and the mouse lymphoma assay, with and without metabolic activation (Seifried et al., 2006[22]). For that reason, significant mutagenic potential is not expected for the structural analogue DEDMSD.

For reproduction toxicity of TMSD, data concerning effects on development are very limited (Doherty et al., 1983[10]), and concerning fertility it is not clear whether or not histopathology of gonads was evaluated in subchronic studies (Johannsen and Levinskas, 1986[17]). For TMPBN and DEDMSD, there are no data concerning reproduction toxicity. Reproduction toxicity, therefore, is a data gap.

Due to structural similarity and due to the comparatively low NOAEL in sub-chronic studies, TMSD was selected for read-across to DEDMSD. Assuming similar toxicodynamic behaviour for TMSD and DEDMSD, both succinodinitriles were compared on the basis of physiology based toxicokinetic modelling. Data used for the PBTK model are summarized in Table 7(Tab. 7).

With these data, the tissue concentration of the substances in man for continuous exposure against 1 mg/m³ was calculated with the program IndusChemFate v2.0 (Jongeneelen and ten Berge, 2011[19]). Data are listed in Table 8(Tab. 8). Based on the calculated results, the toxicokinetic difference between TMSD and DEDMSD is far less than a factor of 2 in VOC target organs.

Therefore, for DEDMSD an equivalent Tentative Indoor Air Guidance Value on a molar basis is proposed as for TMSD, which is 54 µg/m³ or 4.0 x 10^-7^ mol/m³ (Schupp, 2018[21]). This is also the TIAGV for DEDMSD in air on a molar basis, or, on a weight basis,

TIAGV =4.0 x 10^-7^ mol/m³ = 65 µg/m³.

Again, if the phase I metabolism is assumed to be slow for DEDMSD (1^st^ quartile, V_max_/K_m_ = 8.73 h^-1^, Table 1(Tab. 1)) and fast for TMSD (3^rd^ quartile, V_max_/K_m_ = 40.26 Table 1(Tab. 1)), while maintaining the metabolism capacity in the lung, DEDMSD would achieve threefold higher concentrations in the kidneys and about six fold higher concentrations in the liver than TMSD. 

## Discussion

Indoor Air Guidance Values (IAGV) have been derived for those VOCs emitted from polyurethane flexible foam, which up to now have no RW-values (UBA, 2012[24]), LCI-values (Joint Research Center, 2013[18]) or DNEL for consumer (ECHA, 2012[12]). 

For allyloxypropanol (AOP) and diethyl-dimethyl-succinodinitrile (DEDMSD), hardly any toxicological data are available, and read across to structural analogues is required to estimate Indoor Air Guidance Values (IAGV). PBTK modelling was performed to compare equilibrium tissue concentrations for DEDMSD and AOP with their structural analogues, and to find out whether or not additional safety factors have to be introduced due to toxicokinetic differences. 

Metabolic turnover can be introduced in PBTK modelling. For substances without kinetic data, it is always debateable what are the right assumptions for a metabolic turnover. Based on a limited data set of 15 substances, the first, second (= median) and third quartile for hepatic CYP 450 mediated first order reaction rates in liver were estimated with V_max_/K_m_ = 8.73, 15.40 and 40.26 per hour and kg liver. If the target compound has a low turnover rate at the first quartile, while the reference compound has a high turnover rate at the third quartile, the about five times lower rate constant for the target compound results in three to five fold higher concentration in kidney and liver, respectively. It should be noted that the factor between metabolism reaction rates does not automatically result in equivalent factors concerning tissue concentrations. That is, PBTK modelling can not be replaced by simple linear calculations. The easy accessibility of open access software like IndusChemFate should foster the use of PBTK modelling for read across. 

In this work, for read across it was assumed that target compound and structural analogue have the same metabolic rate constants. Only in case of apparently important first metabolites, here the epoxides of allyloxy propanol and bis(allyloxy)butanol, differences in liver CYP 450 on the basis of 1^st^ and 3^rd^ quartiles of V_max_ and K_m_, but with equal EH- and GST-mediated decay rates of the epoxides were assumed to check whether the target compound is prone to generate higher levels of the reactive epoxides against its reference compound. As overall result, the read across factor based on PBTK for allyloxy propanol and diethyl-dimethyl-succinodinitrile is 1. 

This arbitrary approach of metabolism enzymes activity certainly can be challenged, but the limited data set available makes selection of metabolic factors speculative. For the epoxides considered, not only fast epoxidation of their precursor, but certainly also poor EH- and GST-activity would result in elevated tissue levels. Some further in-depth collection and evaluation of available metabolism data is required. Such an exercise may be promising as more robust averages and standard deviations for V_max_ and K_m_ may become available, probably with allocation to certain product groups, p. e. alkenes, aromatics, halogenated compounds etc. With such a database, percentiles might be calculated as a measure for the likelihood for risk estimates for read across. 

Reliability of PBTK supported read across can be improved if for the substance under investigation at minimum physical data like vapour pressure, water solubility, density and Log Kow would be measured. Further improvement can be achieved by the generation of *in vitro* metabolism data; such data could reveal important metabolites and would also provide better estimates for substance turnover. Concerning the substances modelled in this paper, phase I metabolism will probably also generate primary alcohols (supplementary material). Following the action of alcohol and aldehyde dehydrogenases, a certain steady state level of aldehydes may be built up, and aldehyde groups also present reaction sites for proteins and DNA. Modelling of the putative aldehydes was omitted. If modelling of assumed but not proven metabolites is extended, the whole exercise is prone to become complicated and confusing. 

IAGV do not address the odor of compounds. Odor is not regarded as a toxicological endpoint, but it can cause nuisance in residents. For construction products, national regulations to evaluate odor have been imbedded into schemes for the evaluation of construction products (UBA, 2018[25]). Ethers of allylalcohol with propylene glycols were suspected to cause a “musty” odor in polyurethane flexible foams, but it turned out that cyclic acetals of propionaldehyde with propylene glycols were the causative agents (Harris et al., 1988[15]). Concerning other allyloxy-compounds, it is known that allyl-glycidylether has an odor-threshold at 10 ppm (DFG Allyl-glycidylether, 1992[8]); at elevated concentrations, it is a strong irritant to mucous membranes, but at low concentrations the odor was described as not unpleasant. Odor and odor threshold are unknown for DEDMSD and AOP. 

Although PBTK modelling was used, indoor air guidance values should be rated as tentative (TIAGV) when they are based on read across only. TIAGVs should be made use of only as a temporal benchmark, and they should be replaced by IAGV especially for those VOCs, which are detected frequently and/or at high concentrations indoors. 

## Acknowledgements

This research was funded by EUROPUR. The views and interpretations presented are those of the author and not necessarily those of EUROPUR.

## Declaration

The author worked until 2012 for BASF, a major producer of polyurethane flexible foam raw materials.

## Supplementary Material

Supplementary material

## Figures and Tables

**Table 1:**
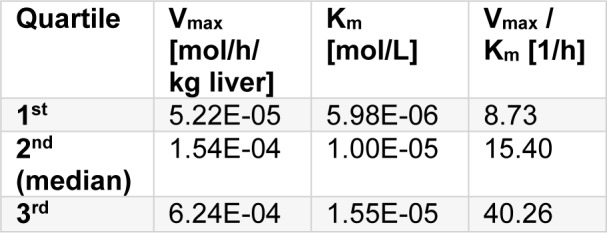
Quartiles for human liver CYP metabolism in the PBTK modelling

**Table 2:**
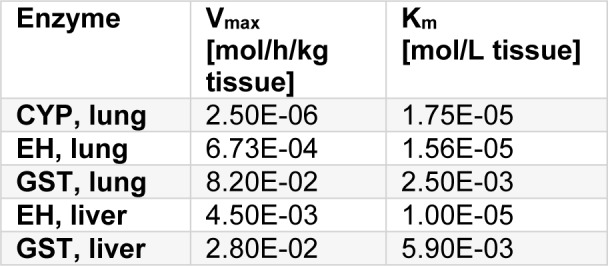
Activities of CYP in lung, EH and GST in lung and liver (Csanady et al., 1994, 2003)

**Table 3:**
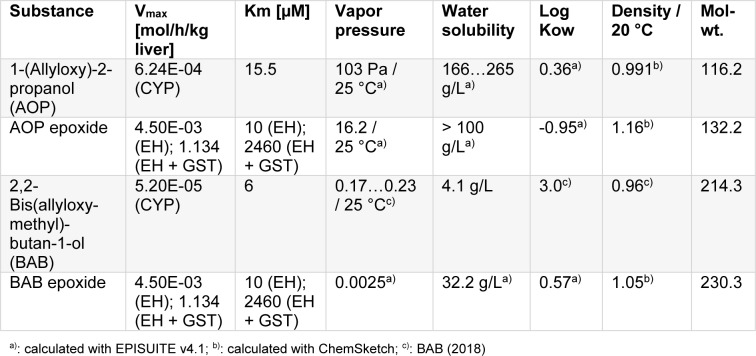
Physical chemical data and enzymatic data for PBTK modelling

**Table 4:**
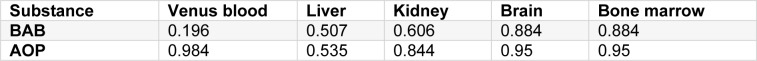
Calculated steady state concentrations in tissues [µmol/L] for AOP and BAB, respectively, against 1 mg/m³ air exposure.

**Table 5:**
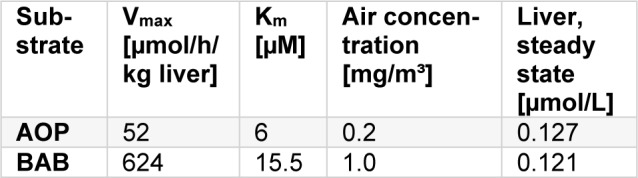
Steady state concentrations of AOP and BAB in liver in dependence on CYP 2E1 activity and air concentration

**Table 6:**
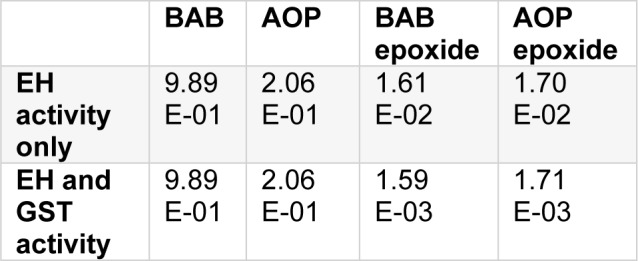
Levels of BAB and AOP and their epoxides in human liver [µmol/L] after continuous exposure against 1 mg/m²

**Table 7:**
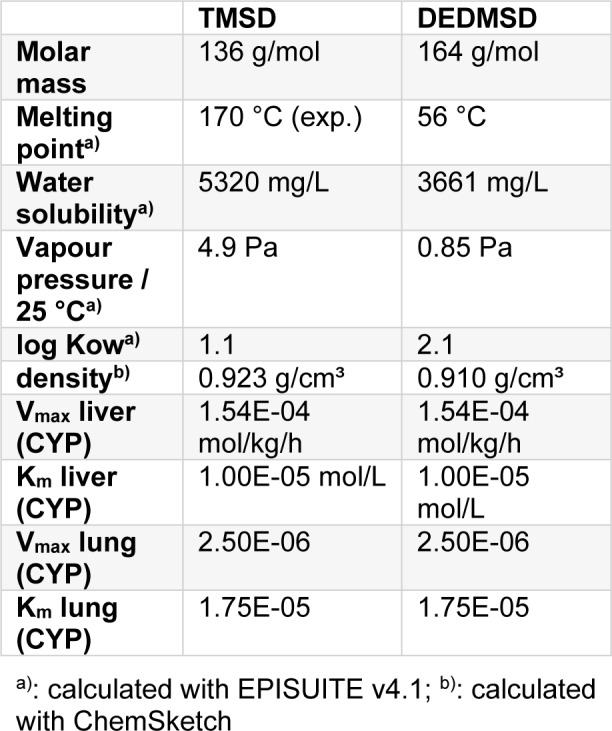
Physical and enzyme kinetic data for tetramethyl-succinodinitrile (TMSD) and 2,3-diethyl-2,3-dimethyl-succinodinitrile (DEDMSD)

**Table 8:**
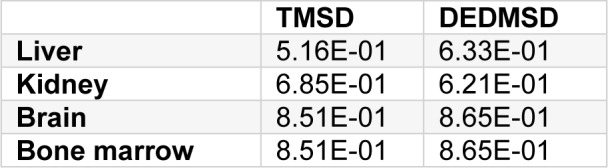
Calculated steady state concentrations [µmol/L] of TMSD and DEDMSD in different tissues following inhalation exposue against 1 mg/ m³
