# Supplementary material for: Read across for the derivation of Indoor Air Guidance Values supported by PBTK modelling
Source: EXCLI J. 2018 Nov 5;17:1069–78. doi: 10.17179/excli2018-1734 (PMC6295638; doi:10.17179/excli2018-1734)
Supplement: Supplementary material [file EXCLI-17-1069-s-001.pdf]

EXCLI Journal 2018;17:1069-1078 – ISSN 1611-2156
Received: September 20, 2018, accepted: October 24, 2018, published: November 05, 2018

Supplementary material to:

READ ACROSS FOR THE DERIVATION OF INDOOR AIR GUIDANCE VALUES SUPPORTED BY PBTK MODELLING

Thomas Schupp

Muenster University of Applied Science, Chemical Engineering, Stegerwaldstrasse 39, D-48565 Steinfurt, GERMANY, Tel.: +49 2551 962595,
E-mail: thomas.schupp@fh-muenster.de

http://dx.doi.org/10.17179/excli2018-1734

This is an Open Access article distributed under the terms of the Creative Commons Attribution License (http://creativecommons.org/licenses/by/4.0/).

PHYSIOLOGY BASED TOXICOKINETIC MODELLING (PBTK)

CYP activities in human liver

A cursory literature search for CYP 450 activity in liver was run in TOXLINE (https://toxnet.nlm.nih.gov/newtoxnet/toxline.htm) searching for “CYP”, “human”, “liver”, “V_{max}” and “K_m”. Data found are summarized in Table A1.

Table A1: Data for human hepatic metabolism (CYP) for some VOC

Substrate	Vmax [M/h]	Km [M]
**Chlorzoxazon**[a)]	7.43E-04	2.75E-04
**p-Nitrophenol**[a)]	1.54E-04	1.29E-04
**Vinylfluoride**[b)]	4.11E-05	4.35E-07
**Aceton**[c)]	1.49E-05	8.60E-07
**Styrene**[d)]	2.00E-03	1.00E-05
**Pyrene**[e)]	3.60E-04	4.50E-06
**tert-Butyl-methylether**[e)]	5.00E-04	5.00E-05
**n-Heptane**[e)]	1.40E-02	1.10E-03
**Trichloro-ethylene**[f)]	3.38E-05	1.33E-05
**Acrylonitrile**[g)]	6.24E-04	1.55E-05
**Chloroform**[h)]	1.29E-04	3.50E-06
**Toluene**[i)]	5.22E-05	5.98E-06
**Ethylbenzene**[i)]	6.89E-05	1.29E-05
**Xylene**[i)]	5.19E-05	2.08E-06
**Benzene**[j)]	2.04E-04	4.49E-06

[a)]: Hanioka et al. (2003); [b)]: Cantoreggi and Kellner (1997); [c)]: Kumagai and Matsunaga (1995); [d)]: Csanady et al. (1994, 2003); [e)]: Jongeneelen and ten Berge (2011); [f)]: Fisher (2000); [g)]: Kedderis et al. (1993); [h)]: Webster et al. (2016); [i)]: Tardif et al. (1997); [j)]: Travis et al. (1990);

At low VOC concentrations the Michaelis-Menten kinetic is expected to be first order, as expressed by the ratio V_{max} / K_m, which is a first order reaction rate constant. The difference in this reaction rate for CYP metabolism (V_{max}/K_m), according to Table 1, is up to 168. For the read across between substances based on the PBTK model IndusChemFate v2.1, the median was used for V_{max} and K_m (Table A2).

Table A2: Quartiles for human hepatic CYP metabolism in the PBTK modelling

Quartile	V_{max} [M/h]	K_m [M]
**1st**	5.22E-05	5.98E-06
**2nd (median)**	1.54E-04	1.00E-05
**3rd**	6.24E-04	1.55E-05

For read across purposes it is generally accepted that the toxic action of a compound is driven by its concentration over time in the target organ – the toxicokinetic factor – and its specific interaction with the target tissue – the toxicodynamic factor. The toxicokinetic factor can be addressed by PBTK modelling. If specific data for a substance are missing it is always critical what is the right assumption for a metabolic turnover. In this work, it is assumed that the target compound and its reference compound are not different against CYP 450 mediated reactions. However, to demonstrate potential critical issues it is assumed that the target compound may show a CYP 450 mediated turnover at the third quartile and the reference compound having an activity at the first quartile, or vice versa, whatever would represent the worst case.

Propenyloxypropanol (Allyloxypropanol, AOP)

Table A3 below summarizes the physical properties of allyloxypropanols against their read across candidate 2,2-Bis(allyloxy)-2-ethyl-butanol-1- (BAB). These physical data were used to run a PBPK modelling with the free software IndusChemFate v2.0 (Jongeneelen and ten Berge, 2011). The concentration in air was set to 1 mg/m^3, and 28 day non-stop exposure of a man of 70 kg body weight was modelled. In absence of data, enterohepatic circulation was set to zero and renal re-absorption was set to “unknown”. First, concentrations of the respective parent compounds were calculated, assuming an equivalent turnover by phase I metabolism. The assumed CYP 450 activities are given in Table A2.

Table A3: Physical chemical data of AOPs and structural analogues

Substance	CAS-No.	Vapor pressure	Water solubility	Log Kow	Density/ 20 °C	Mol-wt.
**1-(Allyloxy)-2-propanol**	21460-36-6	103 Pa / 25 °C[a)]	166… 265 g/L[a)]	0.36[a)]	0.991[b)]	132.2
**2-(Allyloxy)-1-propanol**	1331-17-5	103 Pa / 25 °C[a)]	166…265 g/L[a)]	0.36[a)]	0.991[b)]	132.2
**2,2-Bis(allyloxymethyl) butan-1-ol**	682-09-7	0.17…0.23 / 25 °C[c)]	4.1 g/L	3.0[c)]	0.96[c)]	214.3

[a)]: calculated with EPISUITE v4.1, EPA (2017); [b)]: calculated with ChemSketch; [c)]: BAB (2018)

If for both compounds, BAB and AOP, the same metabolic turnover in liver is assumed, nearly identical steady state concentrations will be achieved in this organ (Figure A1). However, if BAB is degraded by a rate equivalent to the 3rd quartile of the CYP activity, but AOP is transformed at a 1st quartile rate, only, a five fold smaller air concentration of AOP is required to achieve equal steady state levels in human liver (Figure A2).

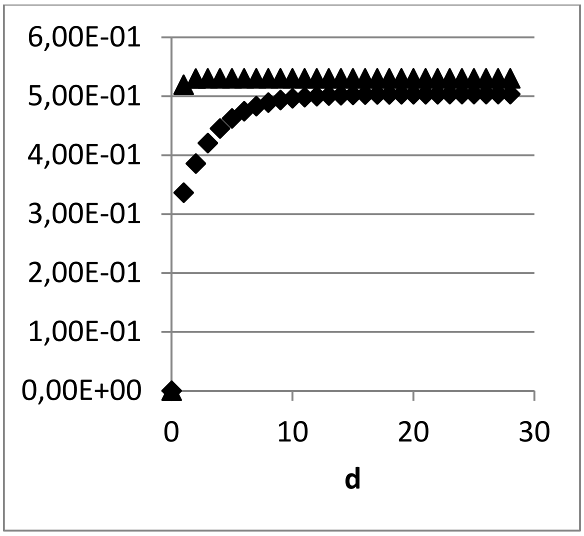

Figure A1: Liver concentration [µmol/L] for V_{max} = 1.54 x 10^{-4} mol/h/kg and K_m = 10^{-5} mol/L for BAB (rhombus) and AOP (triangle) for continuous air exposure against 1 mg/m³

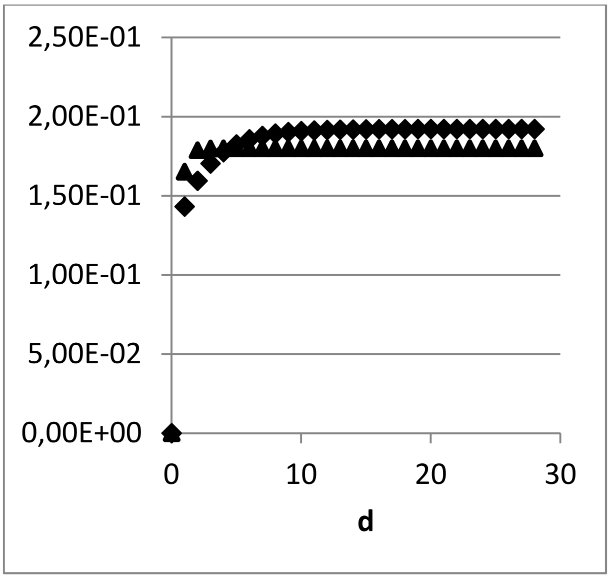

Figure A2: Liver concentration [µmol/L]; BAB (rhombus): C_{air} = 1.0 mg/m³, V_{max} = 6.24 x 10^{-4} mol/h/kg and K_m = 1.6 x 10^{-5} mol/L; AOP (triangles): C_{air} = 0.2 mg/m³, V_{max} = 5.22 x 10^{-5} mol/h/kg and K_m = 5.98 x 10^{-6} mol/L

The phase I metabolism may create epoxides, which can add to biological macromolecules and, therefore, epoxidation is a toxicification. Phase II metabolism may detoxify the epoxides, namely due to hydrolysis catalyzed by epoxyhydrolase (EH) and due to addition of glutathione (GSH), mediated by glutathione-S-transferase (GST).

Enzymatic parameters for these phase II metabolism steps are published by Csanady et al. (1994, 2003) and are summarized in Table A4. The initial concentrations of GSH in liver and lung are 5.9 and 2.0 mM, respectively, and the zero order production rates of GSH in liver and lung are 0.9 and 0.3 mM/h, respectively.

EXCLI Journal 2018;17:1069-1078 – ISSN 1611-2156
Received: September 20, 2018, accepted: October 24, 2018, published: November 05, 2018

Table A4: Enzyme parameters for human lung and liver epoxyhydrolase and glutathione-S-transferase (Csanady et al., 1994, 2003).

	Vmax [M/h]	Km,S [M]	Km,GSH [M]
**GST lung**	8.2E-02	2.5E-03	1.0E-04
**GST liver**	2.8E-02	2.5E-03	1.0E-04
**EH lung**	6.7E-04	1.8E-05	----
**EH liver**	4.5E-03	1.0E-05	----

Epoxide hydrolysis and addition of GSH to epoxides are parallel reactions, both mitigating the epoxide. While the action of epoxide hydrolase can be modelled by simple Michaelis-Menten kinetics, the reaction with glutathione is a bi-substrate reaction with a more complicated rate expression. The decay rate of epoxide is the sum of both reaction rates and can be expressed as

$$-v = \frac{V_{max,EH} \times [S]}{K_{m,EH}+[S]} + \frac{V_{max,GST}\times[GSH]\times[S]}{K_{m,GST,S}[GSH]+K_{m,GST,GSH}[S]+[S][GSH]}$$

(Csanady et al., 1994, 2003). The first term on the right hand side describes the epoxide decay catalyzed by epoxide hydrolase, whereas the second term describes the epoxide reaction with glutathione. This expression is too complicated to introduce it into the convenient IndusChemFate program. However, a few approximations can be introduced. One approximation is the assumption, that GST and GSH don't play a role (worst case), and only epoxide hydrolase serves for the decay of epoxide. The other assumption is that the epoxide concentration is always that low, that the concentration of GSH is not changed and maintained at 2.0 mM in the lung and 5.9 mM in the liver (Csanady et al., 2003). At such conditions it can be assumed that K_m is much larger than the substrate concentration, and the following simplifications are introduced:

$$-v = \frac{V_{max,EH} \times [S]}{K_{m,EH} + [S]} + \frac{V_{max,GST} \times [GSH]_0 \times [S]}{K_{m,GST,S} \times [GSH]_0 + K_{m,GST,GSH} \times [S] + [S] \times [GSH]_0}$$

⬄

$$-v \approx \frac{V_{max,EH} \times [S]}{K_{m,EH}} + \frac{V_{max,GST} \times [GSH]_0 \times [S]}{K_{m,GST,S} \times [GSH]_0 + \{K_{m,GST,GSH} + [GSH]_0\} \times [S]}$$

⬄

$$-v \approx \frac{\{V_{max,EH}\, K_{m,GST,S} + V_{max,GST} K_{m,EH}\} \times [GSH]_0 \times [S]}{K_{m,EH} K_{m,GST,S}[GSH]_0 + K_{m,EH}\{K_{m,GST,GSH} + [GSH]_0\} \times [S]} + \frac{V_{max,EH} \times \{K_{m,GST,GSH} + [GSH]_0\} \times [S]^2}{K_{m,EH} K_{m,GST,S}[GSH]_0 + K_{m,EH}\{K_{m,GST,GSH} + [GSH]_0\} \times [S]}$$

As the second term contains $[S]^2$ while [S] is assumed to be very small, the second term is expected to be much smaller than the first term and, therefore, omitted:

$$-v \approx \frac{\{V_{max,EH}\, K_{m,GST,S} + V_{max,GST} K_{m,EH}\} \times [GSH]_0 \times [S]}{K_{m,EH} K_{m,GST,S}[GSH]_0 + K_{m,EH}\{K_{m,GST,GSH} + [GSH]_0\} \times [S]}$$

Now, the following new constants are introduced,

$$V_{max}^{\#} = \frac{\{V_{max,EH}\ K_{m,GST,S} + V_{max,GST} K_{m,EH}\} \times [GSH]_0}{K_{m,EH}\{K_{m,GST,GSH} + [GSH]_0\}}$$

$$K_m^{\#} = \frac{K_{m,EH} K_{m,GST,S} [GSH]_0}{K_{m,EH}\{K_{m,GST,GSH} + [GSH]_0\}}$$

which results in the rate equation

$$-v = \frac{V_{max}^{\#} \times [S]}{K_m^{\#} + [S]}$$

$V_{max}^{\#}$ and $K_m^{\#}$ can be introduced in the IndusChemFate model, and with respect to the different organs, the values are
for the liver:

$V_{max}^{\#}$ = 1.134 mol/kg/h,
$K_m^{\#}$ = 2.46 x 10^{-3} mol/L,
and for the lung:
$V_{max}^{\#}$ = 1.67 x 10^{-1} mol/kg/h,
$K_m^{\#}$ = 2.38 x 10^{-3} mol/L.

At this point, it is assumed that phase I metabolism is a toxification reaction, whereas phase II metabolism is a detoxification. The steady state concentration of the epoxide following continuous air exposure shall be modelled. Table A5 lists the calculated physico-chemical parameters for the allyloxypropanol (AOP) oxidation product, the Glycidyl-(2-hydroxy)propyl ether (AOP epoxide), and for the 2,2-Bis(Allyloxymethyl)-2-ethyl-butanol-1 (BAB) epoxidation product, the 2-Allyloxymethyl-2-glycidyloxymethyl-2ethyl-butanol-1 (BAB epoxide). As worst case it is assumed that epoxidation of BAB is slow (1st quartile for CYP activity in liver) and AOP epoxidation is fast (3rd quartile CYP acitivity in liver).

Table A5: Physico-chemical data and enzymatic data taken forward for PBTK modelling

Substance	Vmax [M/h]	Km [M]	Vapor pressure	Water solubility	Log Kow	Density / 20 °C	Mol-wt.
**Allyloxy-propanol**	6.24E-04 (CYP, liver); 2.50E-06 (CYP, lung)	15.5 (liver); 17.5 (lung)	103 Pa / 25 °C[a)]	166…265 g/L[a)]	0.36[a)]	0.991[b)]	116.2
**AOP epoxide**	4.50E-03 (EH); 1.134 (EH + GST)	10 (EH); 2460 (EH + GST)	16.2 / 25 °C[a)]	> 100 g/L[a)]	-0.95[a)]	1.16[b)]	132.2
**2,2-Bis(allyloxymethyl)-butan-1-ol (BAB)**	5.20E-05 (CYP, liver); 2.50E-06 (CYP, lung)	6 (liver); 17.5 (lung)	0.17…0.23 / 25 °C[c)]	4.1 g/L	3.0[c)]	0.96[c)]	214.3
**BAB epoxide**	4.50E-03 (EH); 1.134 (EH + GST)	10 (EH); 2460 (EH + GST)	0.0025[a)]	32.2 g/L[a)]	0.57[a)]	1.05[b)]	230.3

[a)]: calculated with EPISUITE v4.1, EPA (2017); [b)]: calculated with ChemSketch v2017.1; [c)]: BAB (2018)

For the AOP epoxide and BAB epoxide, $V_{max}^{\#}$ and $K_m^{\#}$ for liver and lung are introduced in the model, and calculations are run under assumption of constant GSH concentrations. However, the level of GSH may become exhausted, which would finally end up in some cell damage and would not be in concordance with a NOAEL. However, as this exercise is a read

EXCLI Journal 2018;17:1069-1078 – ISSN 1611-2156
Received: September 20, 2018, accepted: October 24, 2018, published: November 05, 2018

across from BAB to AOP, the model is run also under assumption that only epoxide hydrolase serves for the decay of epoxides. Results are shown in Table A6. Naturally, the slower phase I metabolism of BAB compared to AOP results in about five fold higher levels in the liver; decay of the epoxides by EH alone or by EH and GST in combination leads to nearly equivalent levels of epoxides of AOP and BAB, respectively.

Table A6: Levels of BAB and AOP and their epoxides in human liver (µmol/L) after continuous exposure against 1 mg/m²

	BAB	AOP	BAB epoxide	AOP epoxide
**EH activity only**	9.89 E-01	2.06 E-01	1.61 E-02	1.70 E-02
**EH and GST activity**	9.89 E-01	2.06 E-01	1.59 E-03	1.71 E-03

As can be seen in Figure A3, also for the epoxide metabolites BAB takes some more time to achieve the stady state in the liver. In case of GST and EH activity, steady state levels of epoxides in the liver are a factor of about 10 lower when compared to only EH activity. The higher epoxidation rate of AOP against BAB on the one hand causes a five fold lower concentration of the parent compound in the liver, on the other hand a higher concentration of the epoxide in the liver. However, AOP epoxide levels are only 5 to 7 % higher than BAB epoxide levels.

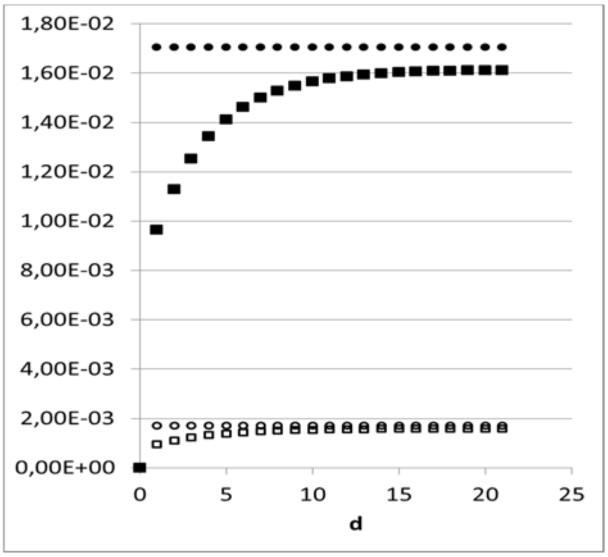

Figure A3: Concentration (µmol/L) of BAB epoxide (squares) and AOP epoxide (circles) depending on EH activity alone (filled symbols) and combined EH and GST activity (empty symbols)

2,3-Diethyl-2,3-dimethy-succinodinitrile (DEDMSD)

For 2,3-diethyl-2,3-dimethyl-succinodinitrile, DEDMSD, two CAS-numbers are available: 128903-20-8 and 85688-81-9. Substance specific toxicological data for DEDMSD were not identified. For read across, data for aliphatic tertiary nitriles were searched. 2,2,3,3,-tetramethyl succino dinitrile (TMSD, CAS-No. 3333-52-6) and 2,2,4-trimethyl-4-phenyl-butane nitrile (TMPBN, CAS-No. 75490-39-0) were detected as structural analogues with repeated dose toxicity data (TMPBN, 2018). To focus the read across on tertiary nitriles is justi-

fied as primary and secondary nitriles may undergo alpha-hydroxylation; the resulting cyanohydrins can easily release cyanide ions, which is not the case for the tertiary nitriles. For example, sodium thiosulfate as a sulfur depot for detoxification of cyanide was protective for intoxication with adiponitrile by supporting the conversion of cyanide to thiocyanate, but for TMSD it was without any effect (Doherty et al., 1982).

Both succinodinitriles were compared on the basis of physiology based toxicokinetic modelling. With these data, the blood concentration of the substances in man for continuous exposure against 1 mg/m³ was calculated with the program IndusChemFate v2.0 (Jongeneelen and ten Berge, 2011). Data are shown in Table A7. Based on the calculated results, the toxicokinetic difference between TMSD and DEDMSD is far less than a factor of 2 in typical VOC target organs brain, liver and kidneys.

Again, it may be assumed that the CYP 450 metabolism of TMSD is fast (3rd quartile, Table A2) and that of DEDMSD is slow (1st quartile, Table A2). Figure A4 shows the concentration in the target organ liver in dependence on exposure time; Figure A5 shows the concentration-time curve for the target organ kidney.

Table A7: Physico-chemical and enzyme kinetic data for tetramethyl succinodinitrile (TMSD) and 2,3-diethyl-2,3-dimethyl-succinodinitrile (DEDMSD)

	**TMSD**	**DEDMSD**
**Molar mass**	136 g/mol	164 g/mol
**Melting point[a)]**	170 °C (exp.)	56 °C
**Water solubility[a)]**	5320 mg/L	3661 mg/L
**Vapour pressure / 25 °C[a)]**	4.9 Pa	0.85 Pa
**log Kow[a)]**	1.1	2.1
**density[b)]**	0.923 g/cm³	0.910 g/cm³
**V_{max} liver (CYP)**	1.54E-04 mol/kg/h	1.54E-04 mol/kg/h
**K_m liver (CYP)**	1.00E-05 mol/L	1.00E-05 mol/L
**V_{max} lung (CYP)**	2.50E-06	2.50E-06
**K_m lung (CYP)**	1.75E-05	1.75E-05

[a)]: calculated with EPISUITE v4.1, EPA (2017); [b)]: calculated with ChemSketch

Table A8: Calculated steady state concentrations [µmol/L] of TMSD and DEDMSD in different tissues following continuous exposure against 1 mg/m³

	**TMSD**	**DEDMSD**
**Venous blood**	0.726	0.407
**Liver**	0.554	0.672
**Kidney**	0.735	0.659
**Brain**	0.912	0.918

EXCLI Journal 2018;17:1069-1078 – ISSN 1611-2156
Received: September 20, 2018, accepted: October 24, 2018, published: November 05, 2018

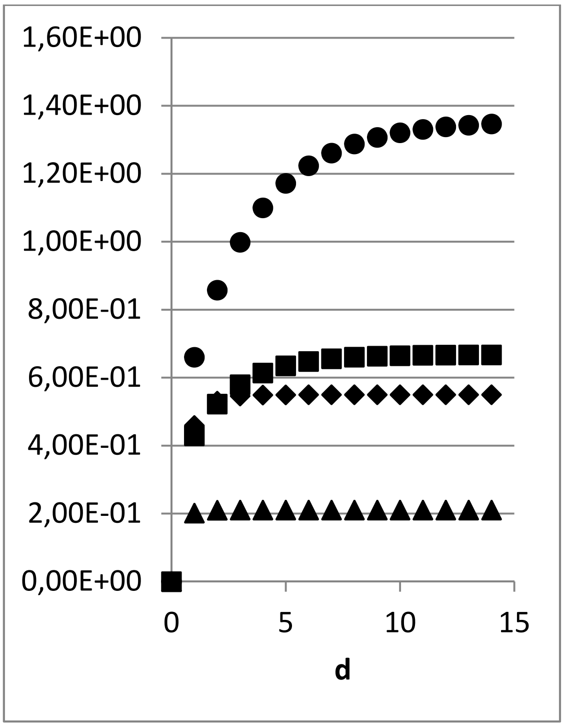

Figure A4: Liver concentration (µmol/L): TMDS at median (rhombus) or 3rd quartile (triangle) CYP activity; DEDMSD at median (squares) and 1st quartile (circles) CYP activity

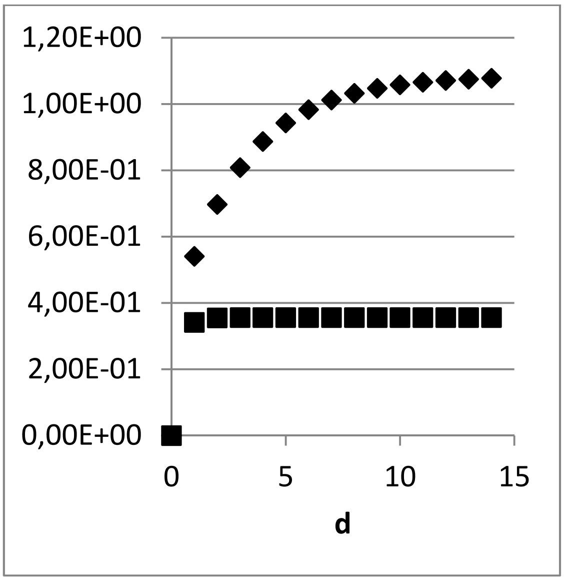

Figure A5: Steady state kidney concentration (µmol/L) of TMSD (squares; CYP 3rd = quartile) and DEDMSD (rhombus, CYP = 1st quartile)

Therefore, if the turnover of TMSD in liver is high (3rd quartile) and that of DEDMSD is low (1st quartile), an air concentration of 0.18 mg/m³ DEDMSD would result in an equivalent steady state liver and kidney concentrations as 1 mg/m³ TMSD.

EXCLI Journal 2018;17:1069-1078 – ISSN 1611-2156
Received: September 20, 2018, accepted: October 24, 2018, published: November 05, 2018

METBOLITE PREDICTIONS

Metabolites were predicted with help of the OECD QSAR Toolbox (2018), version 4.2, with the rat *in vivo* metabolism simulator.

Metabolites of Allyloxypropanol

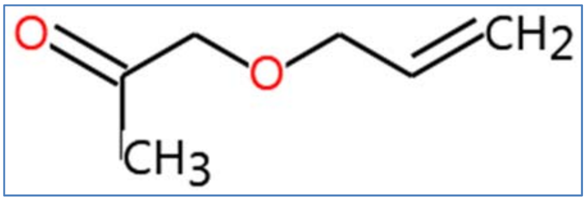

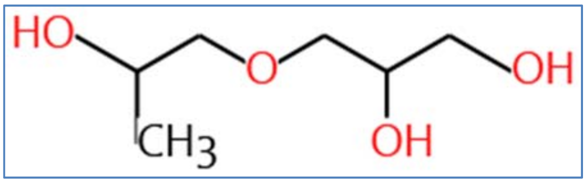

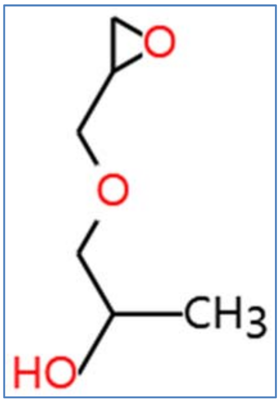

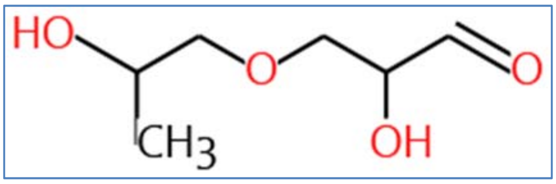

EXCLI Journal 2018;17:1069-1078 – ISSN 1611-2156
Received: September 20, 2018, accepted: October 24, 2018, published: November 05, 2018

Metabolites of 2,2-Bis(allyloxymethyl)-butan-1-ol (BAB)

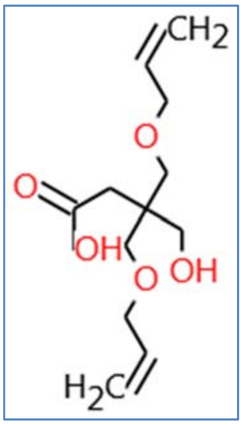

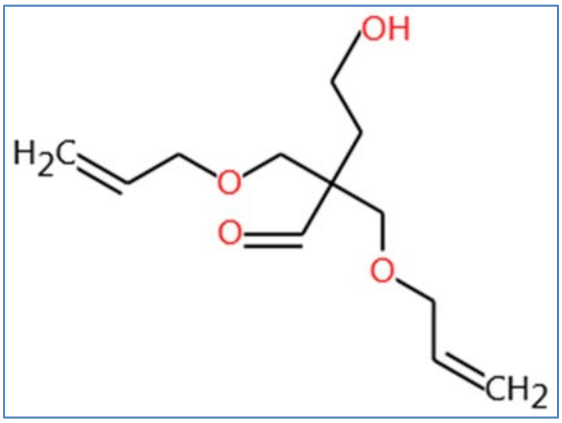

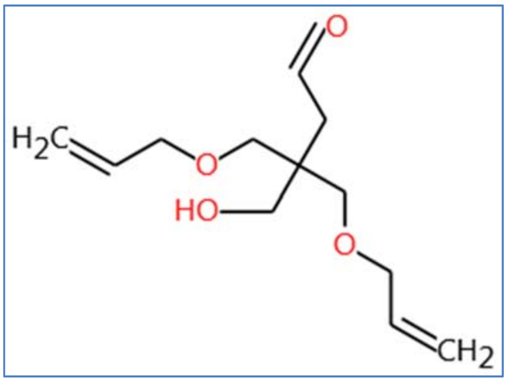

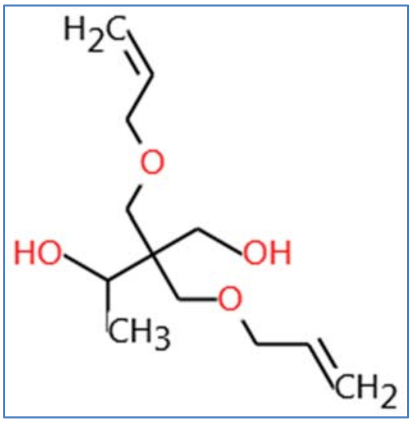

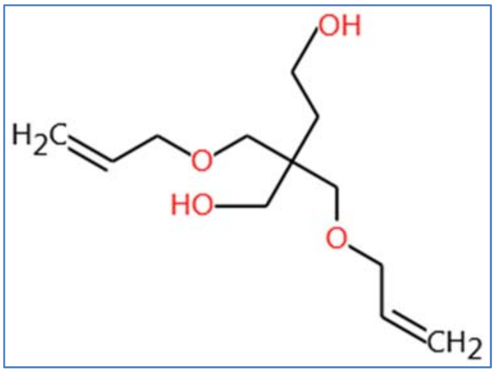

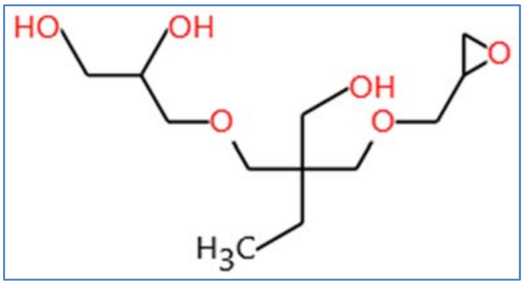

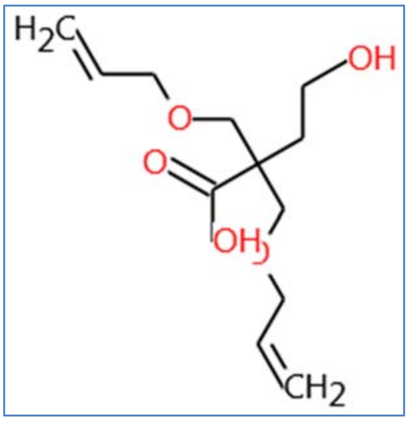

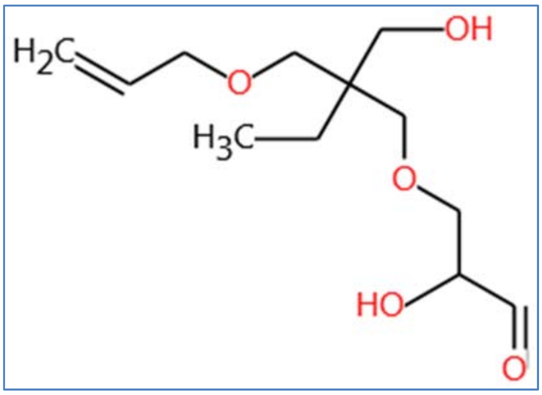

EXCLI Journal 2018;17:1069-1078 – ISSN 1611-2156
Received: September 20, 2018, accepted: October 24, 2018, published: November 05, 2018

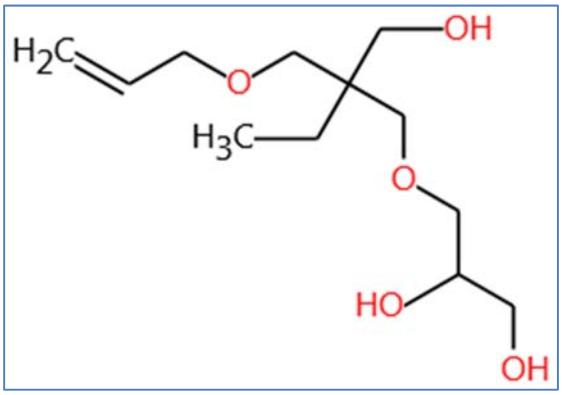

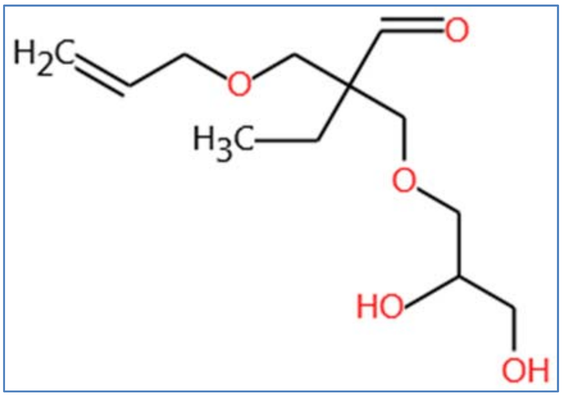

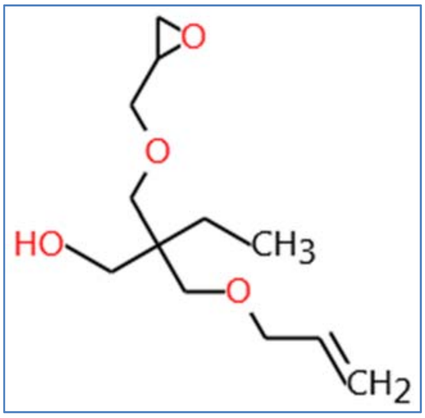

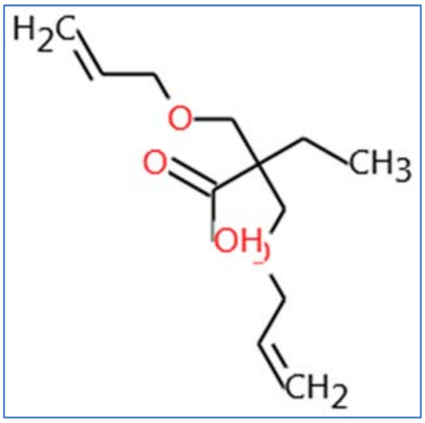

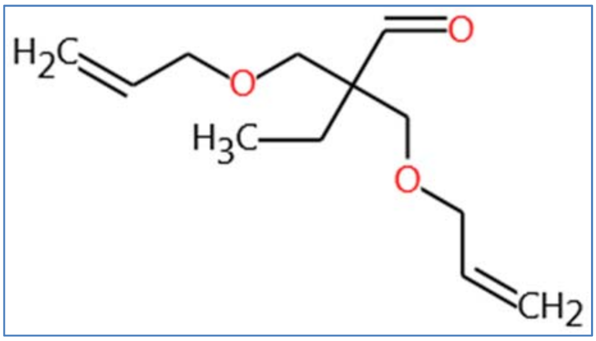

Metabolites of 2,3-diethyl-2,3-dimethyl-succinodinitrile (DEDMSD)

In vivo rat metabolism simulator results:

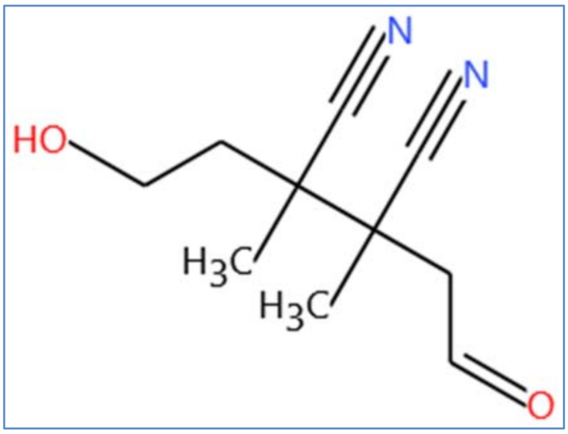

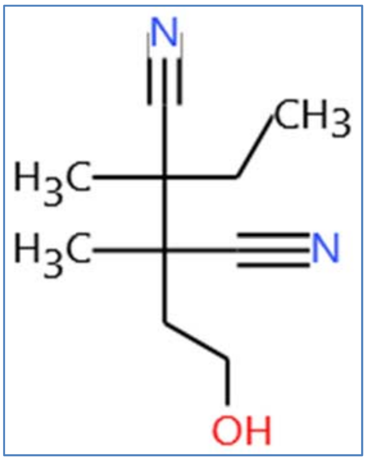

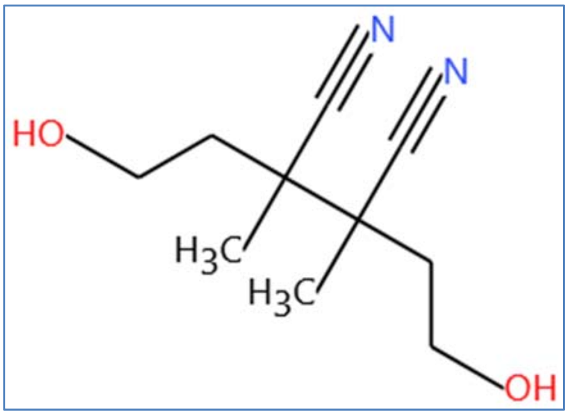

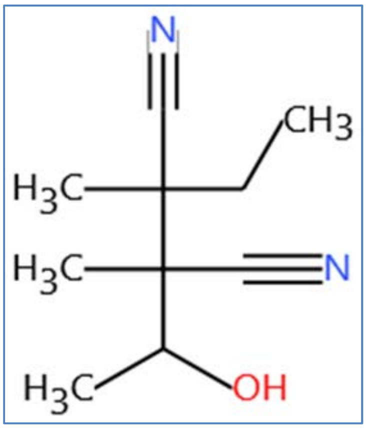

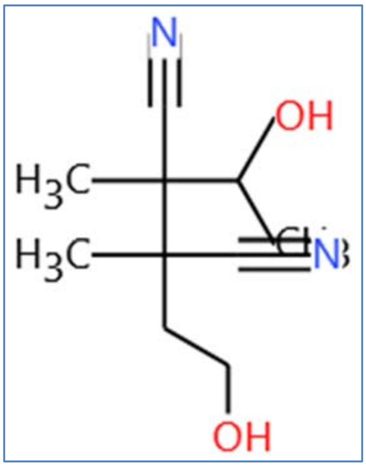

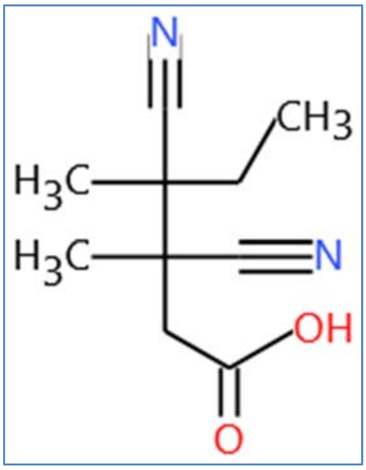

EXCLI Journal 2018;17:1069-1078 – ISSN 1611-2156
Received: September 20, 2018, accepted: October 24, 2018, published: November 05, 2018

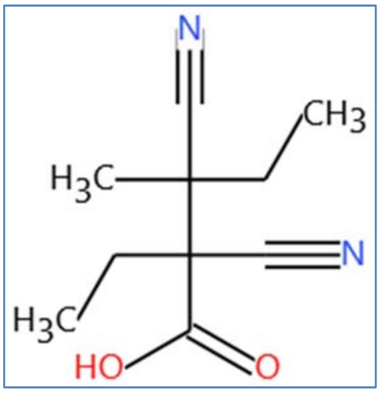

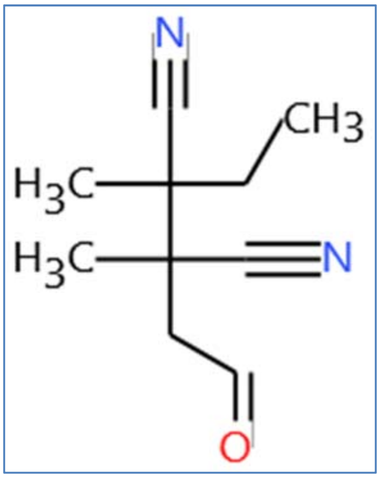

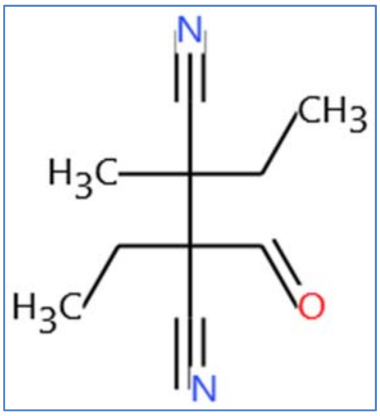

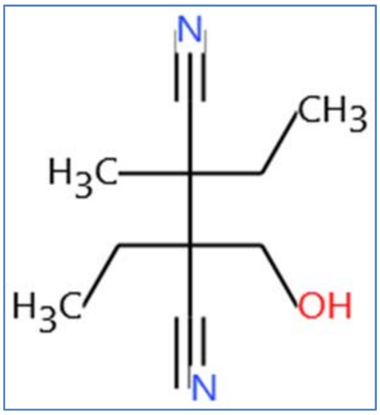

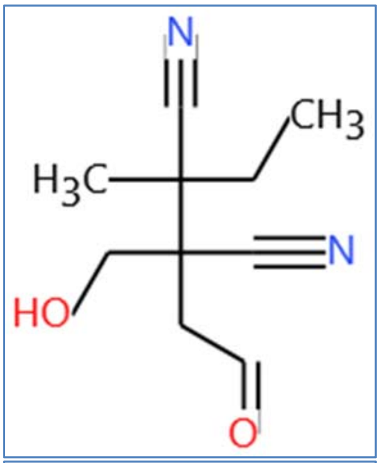

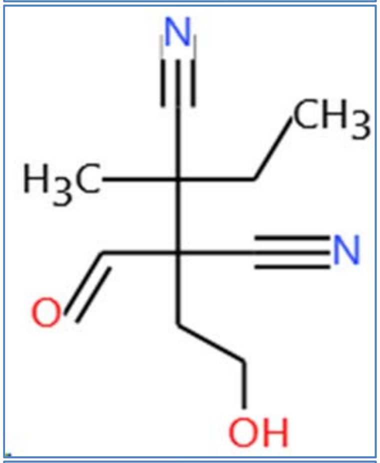

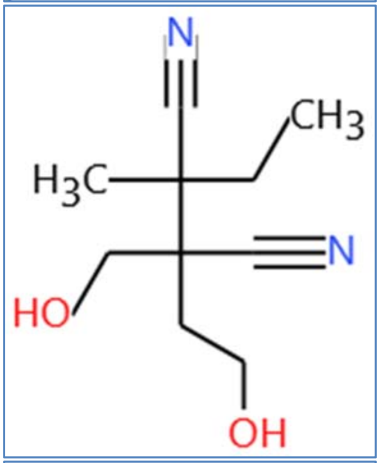

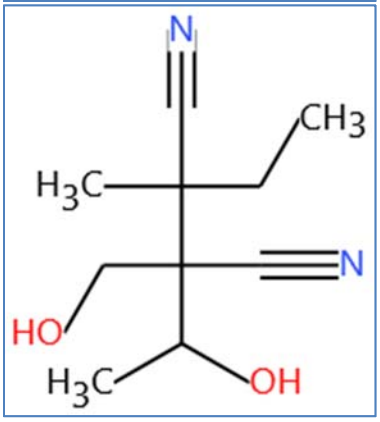

EXCLI Journal 2018;17:1069-1078 – ISSN 1611-2156
Received: September 20, 2018, accepted: October 24, 2018, published: November 05, 2018

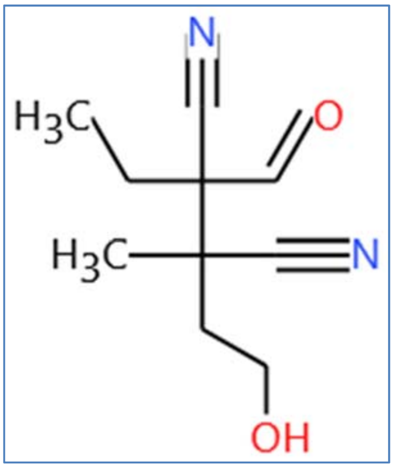

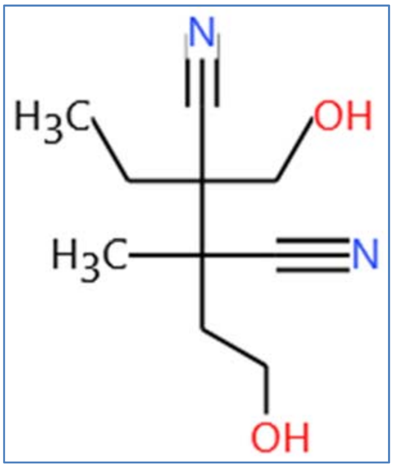

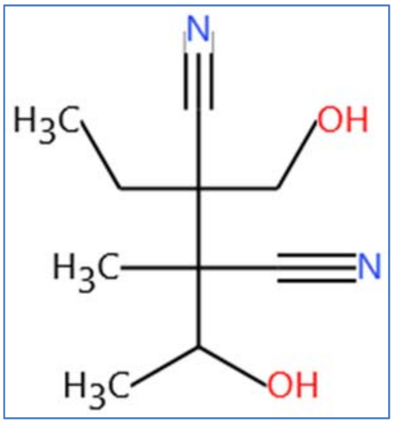

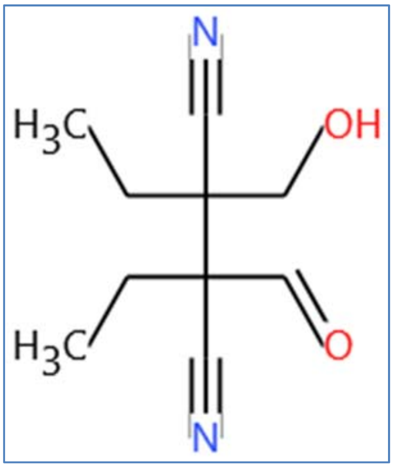

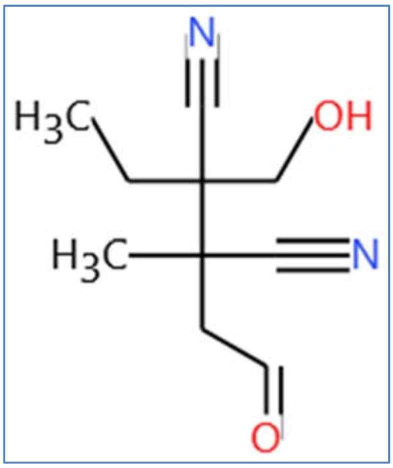

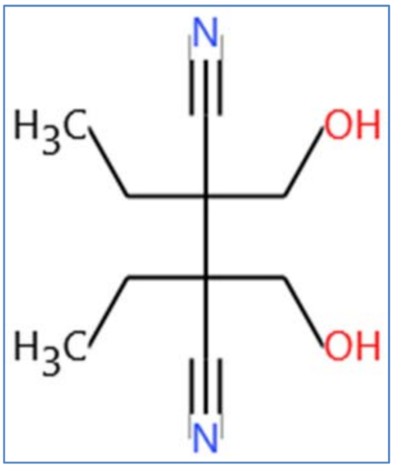

EXCLI Journal 2018;17:1069-1078 – ISSN 1611-2156
Received: September 20, 2018, accepted: October 24, 2018, published: November 05, 2018

Metabolites of 2,2,3,3-tetramethyl-succinodinitrile (TMSD)

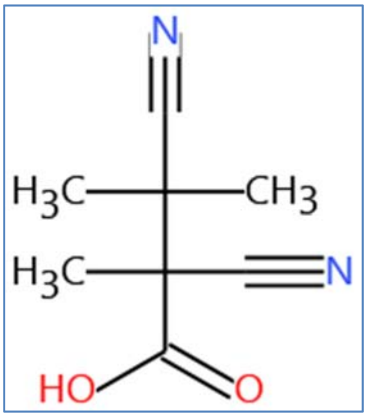

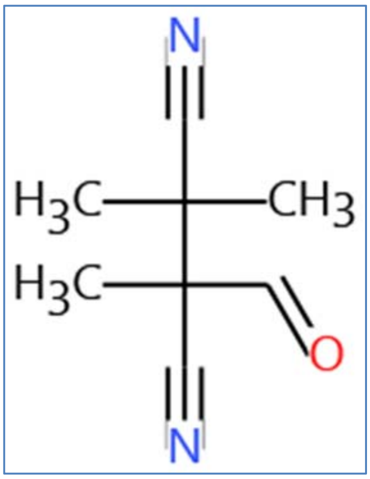

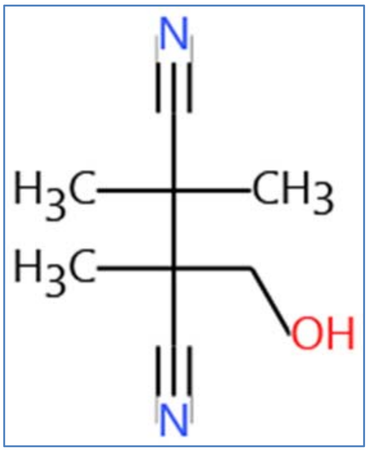

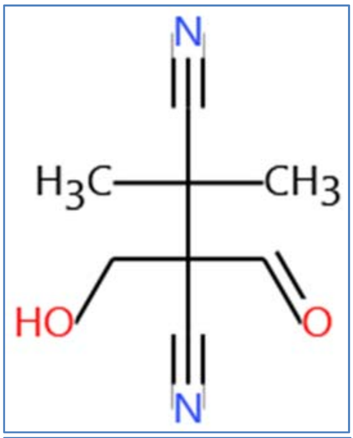

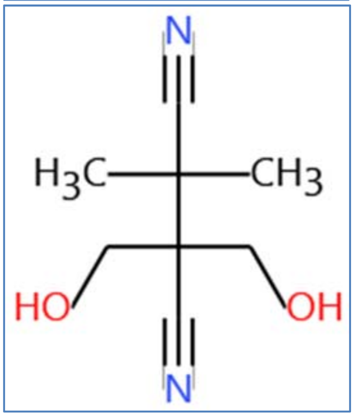

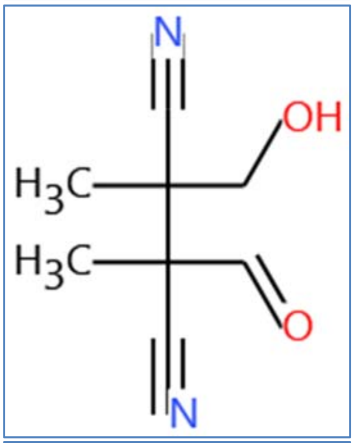

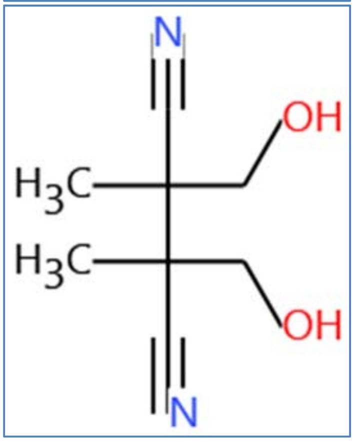

EXCLI Journal 2018;17:1069-1078 – ISSN 1611-2156
Received: September 20, 2018, accepted: October 24, 2018, published: November 05, 2018

REFERENCES

AEPD. Registration Dossier 2-Allyloxy-2-ethylpropanediol-1,3. European Chemicals Agency, 2018. https://echa.europa.eu/de/registration-dossier/-/registered-dossier/10272. Accessed 06 September 2018.

BAB. Registration Dossier 2,2-Bis(allyloxymethyl)butan-1-ol. European Chemicals Agency, 2018. https://echa.europa.eu/de/registration-dossier/-/registered-dossier/1894. Accessed 06 September 2018.

Cantoreggi S, Kellner DA. Pharmacokinetics and metabolism of vinylfluoride in vivo and in vitro. Toxicol Appl Pharmacol. 1997;143:130-9.

Csanady GA, Mandrala AL, Filser JG. A physiologic pharmacokinetic model for styrene and styrene-7,8-oxide in mouse, rat and man. Arch Toxicol. 1994;68: 143-57.

Csanady GA, Kessler W, Hoffmann HD, Filser JG. A toxicokinetic model for styrene and ist metabolite styrene-7,8-epoxide in mouse, rat and human with special emphasis on the lung. Toxicol Lett. 2003;138:75-102.

Doherty PA, Smith RP, Ferm VH. Tetramethyl substitution on succinonitrile confers pentylenetetrazol-like activity and blocks cyanide release in mice. J Pharmacol Exp Ther. 1982;223:635-41.

EPA. EPISUITE v.4.1. U.S. Environmental Protection Agency. https://www.epa.gov/tsca-screening-tools/download-epi-suitetm-estimation-program-interface-v411. Accessed 15 May 2017.

Fisher JF. Physiologically based pharmacokinetic models for trichloroethylene and its oxidative metabolites. Environ Health Perspect. 2000;108(S2):249-55.

Hanioka N, Tanaka-Kagawa T, Miyata Y, Matsushima E, Makino Y, Ohno A, et al. Functional characterization of three cytochrome P450 2E1 variants with amino acid substituents. Xenobiotica. 2003;33:575-86.

Jongeneelen FJ, ten Berge W. A generic, cross-chemical predictive PBTK model with multiple entry routes running as application in MS Excel; design of the model and comparison of predictions with experimental results. Ann Work Expos Health. 2011;55:841-64. http://cefic-lri.org/toolbox/induschemfate/. Accessed 04 September 2018.

Kedderis GL, Batra R, Koop PR. Epoxidation of acrylonitrile by rat and human cytochrome P450. Chem Res Toxicol. 1993;6:866-71.

Kumagai S, Matsunaga I. Physiologicxally based pharmacokinetic model for acetone. Occup Environ Med. 1995;52:344-52.

OECD. QSAR Toolbox. https://www.qsartoolbox.org/.; Accessed 28 August 2018.

Tardif R, Charest-Tardif G, Brodeur J, Krishnan K. Physiologically based pharmacokinetic modeling of a ternary mixture of alkylbenzenes in rats and humans. Toxicol Appl Pharmacol. 1997;144:120-34.

TMPBN. Registration Dossier 2,2,4-trimethyl-4-phenyl-butane nitrile; European Chemicals Agency, 2018. https://echa.europa.eu/de/registration-dossier/-/registered-dossier/1457. Accessed 17 September 2018.

Travis CC, Quillen JL, Arms AD. Pharmacokinetics of benzene. Toxicol Appl Pharmacol. 1990;102:400-20.

Webster EM, Qian H, Mackay D, Christensen RD, Tietjen B, Zaleski R. Modeling human exposure to indoor contaminants: external source to body tissue. Environ Sci Technol. 2016;50:8697-704.
